# Aromatic Residues on the Side Surface of Cry4Ba-Domain II of *Bacillus thuringiensis* subsp. *israelensis* Function in Binding to Their Counterpart Residues on the *Aedes aegypti* Alkaline Phosphatase Receptor

**DOI:** 10.3390/toxins15020114

**Published:** 2023-01-29

**Authors:** Anon Thammasittirong, Sutticha Na-Ranong Thammasittirong

**Affiliations:** 1 Department of Microbiology, Faculty of Liberal Arts and Science, Kasetsart University, Nakhon Pathom 73140, Thailand; 2 Microbial Biotechnology Unit, Faculty of Liberal Arts and Science, Kasetsart University, Nakhon Pathom 73140, Thailand

**Keywords:** *Aedes aegypti*, *Bacillus thuringiensis*, Cry4Ba toxin, dengue fever, receptor binding

## Abstract

Receptor binding is a prerequisite process to exert the mosquitocidal activity of the Cry4Ba toxin of *Bacillus thuringiensis* subsp. *israelensis*. The beta-sheet prism (domain II) and beta-sheet sandwich (domain III) of the Cry4Ba toxin have been implicated in receptor binding, albeit the precise binding mechanisms of these remain unclear. In this work, alanine scanning was used to determine the contribution to receptor binding of some aromatic and hydrophobic residues on the surface of domains II and III that are predicted to be responsible for binding to the *Aedes aegypti* membrane-bound alkaline phosphatase (Aa-mALP) receptor. Larvicidal activity assays against *A. aegypti* larvae revealed that aromatic residues (Trp_327_ on the β2 strand, Tyr_347_ on the β3–β4 loop, and Tyr_359_ on the β4 strand) of domain II were important to the toxicity of the Cry4Ba toxin. Quantitative binding assays using enzyme-linked immunosorbent assay (ELISA) showed similar decreasing trends in binding to the Aa-mALP receptor and in toxicity of the Cry4Ba mutants Trp327Ala, Tyr347Ala, and Tyr359Ala, suggesting that a possible function of these surface-exposed aromatic residues is receptor binding. In addition, binding assays of the Cry4Ba toxin to the mutants of the binding residues Gly_513_, Ser_490_, and Phe_497_ of the Aa-mALP receptor supported the binding function of Trp_327_, Tyr_347_, and Tyr_359_ of the Cry4Ba toxin, respectively. Altogether, our results showed for the first time that aromatic residues on a side surface of the Cry4Ba domain II function in receptor binding. This finding provides greater insight into the possible molecular mechanisms of the Cry4Ba toxin.

## 1. Introduction

Dengue infection, causing dengue fever, dengue hemorrhagic fever (DHF), and dengue shock syndrome (DSS), is a major global public health concern [1]. Dengue is a mosquito-borne *Flavivirus* infection that is primarily transmitted by *Aedes aegypti* [2]. Dengue is hyperendemic in tropical and subtropical regions and has been reported in more than 100 countries [2,3]. Globally, there were an estimated 390 million infections in 2010, of which 96 million cases had apparent clinical symptoms [4]. The geographical range of dengue is expected to expand into low-risk or dengue-free areas due to climate change, with approximately 6.1 billion people (60% of the world’s population) predicted to be at risk of dengue infection in 2080 [5]. Currently, only one commercially available vaccine against dengue viruses (DENVs) has been developed and licensed under the trade name Dengvaxia. According to the World Health Organization (WHO) in 2018, the Dengvaxia vaccine has been recommended only for use with dengue-seropositive persons aged 9–45 years or for individuals in areas with a high seroprevalence of dengue [6]. However, due to concerns about the low effectiveness and the side effects of this vaccine, many people have been reluctant to be vaccinated [7]. Therefore, mosquito vector control remains an important strategy to prevent dengue outbreaks.

*Bacillus thuringiensis* subsp. *israelensis* (*Bti*) is one outstanding microbial insecticide that has been recommended by the WHO to control the *Aedes* vector [1]. *Bti*, a Gram-positive endospore-forming bacterium, produces a set of four major insecticidal toxins—Cry4Aa (~125 kDa), Cry4Ba (~130 kDa), Cry11Aa (~68 kDa), and Cyt1Aa (~28 kDa)—forming as parasporal crystalline inclusion bodies with high specific toxicity to some dipteran insect larvae, including mosquitoes and black flies [8,9]. *Bti* has proven effectiveness as it has been used to control mosquitoes for decades without any resistance reported in mosquito field populations [10,11]. On the laboratory scale, a high resistance level to a single *Bti* toxin has been reported in several mosquito species; however, no or very low resistance levels have been reported after 30 generations of exposure to *Bti* or its four mixture toxins [11,12]. The lack of resistance to *Bti* after long-term use is due to synergistic effects among the synthesized toxins, especially synergism between the Cyt1A and Cry toxins [9,13]. Although *Bti* has been used to control mosquito vectors for several decades, the precise mechanisms of its toxins remain to be explored.

The X-ray crystallographic structure of the Cry4Ba toxin revealed a high degree of structural conservation to other Cry toxins [14], indicating that they share a common mode of action to larvicidal activity. The N-terminal domain I is composed of a seven-α-helix bundle function in a midgut pore formation while the β-prism domain II and the β-sandwich domain III are involved in receptor binding (for a review, see [9]). A number of *A. aegypti* midgut proteins have been reported to interact with the Cry4Ba toxin [15]; among these, glycosylphosphatidylinositol (GPI)-anchor alkaline phosphatase (ALP) and aminopeptidase N (APN) have been well characterized as receptors for the Cry4Ba toxin [16,17,18,19,20]. In our recent study, we provided evidence that both the full-length Cry4Ba toxin and its isolated domain III could bind to the membrane-bound ALP (Aa-mALP) receptor from *A. aegypti* larvae [21].

**Figure 1:**
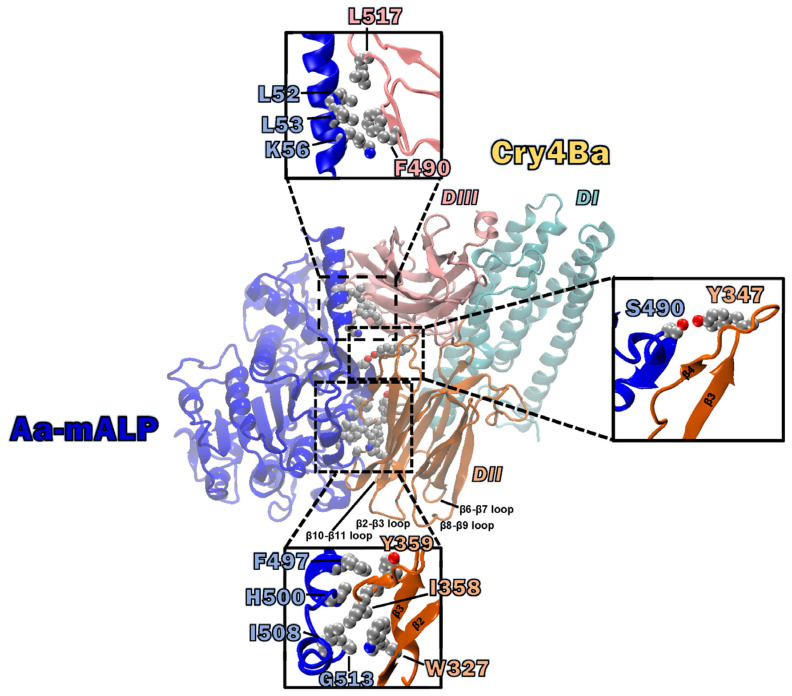
Ribbon representation of docking conformation of Cry4Ba (domain I (DI): green; domain II (DII): orange; domain III (DIII): pink) and the Aa-mALP receptor (blue) prepared using VMD software [23]. Ball and stick models represent some potential binding residues (aromatic and hydrophobic side chains of domain II (Trp_327_, Tyr_347_, Ile_358_, and Tyr_359_) and domain III (Phe_490_ and Leu_517_) of the Cry4Ba toxin along with their counterparts on the Aa-mALP receptor).

Several surface-exposed amino acid residues, such as Thr_386_, Ser_387_, Ser_388_, Pro_389_, Ser_390_, and Asn_391_ on the β6-β7 loop, Glu_417_ on the β8-β9 loop, and Tyr_455_ and Asn_456_ on the β10-β11 loop, at the bottom of Cry4Ba domain II have been reported as important for toxicity against *A. aegypti* larvae [16,22]. Mutation of these residues affected the binding of Cry4Ba to *A. aegypti* midgut brush border membrane vesicles (BBMV) [16,22] or the purified ALP1 isoform receptor [16]. In a recent study using molecular docking interaction and site-specific mutagenesis, we have shown that Leu_615_ on the β22–β23 loop of Cry4Ba-DIII was involved in binding to the purified Aa-mALP receptor and related to toxicity against *A. aegypti* larvae [21]. However, other residues of the Cry4Ba domains DII and III predicted by a molecular docking study as binding residues, especially aromatic and hydrophobic side chains, remain to be explored. Therefore, the present work performed further characterizations for the binding relevance of some aromatic and hydrophobic residues on a surface of domain II (Trp_327_, Tyr_347_, Ile_358_, and Tyr_359_) and domain III (Phe_490_ and Leu_517_) on a side of the Cry4Ba toxin predicted as binding residues and for their counterpart residues (Gly_513_, Ser_490_, His_500,_ Phe_497_, Leu_53_, and Leu_52_, respectively) on Aa-mALP (Figure 1).

## 2. Results

### 2.1. Analysis of Possible Binding Residues between the Cry4Ba Toxin and the Aa-mALP Receptor

**Figure 2:**
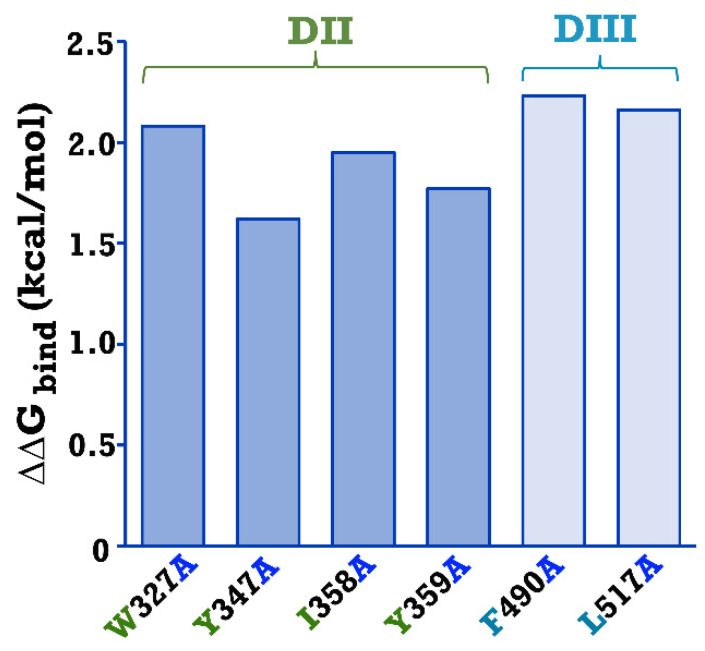
Changes in binding free energy (ΔΔG_bind_) in the Cry4Ba—Aa-mALP complex following alanine substitution of the selected Cry4Ba residues analyzed using BeAtMuSiC approach [24], where ΔΔG_bind_ > 0 means decreased binding affinity.

Analysis of the molecular docking interaction between the Cry4Ba toxin and the Aa-mALP receptor revealed that the Cry4Ba toxin used the surface residues of both domains II and III on a side of the molecule to bind to the Aa-mALP receptor (Figure 1). The docking result showed that the major domain II residues that function in binding to the Aa-mALP receptor are on a side surface rather than only as residues on the beta-hairpin loops located on the underside of the toxin. In our previous work, some binding residues from this docking result were studied and we found that Leu_615_ in the β22–β23 loop of the Cry4Ba domain III toxin was a crucial residue for Aa-mALP receptor binding [21]. In the present study, aromatic and hydrophobic residues of Cry4Ba domain II (Trp_327_, Tyr_347_, Ile_358_, and Tyr_359_) and domain III (Phe_490_ and Leu_517_) and their counterpart interacting residues (Gly_513_, Ser_490_, His_500,_ Phe_497_, Leu_53_, and Leu_52_, respectively) on Aa-mALP (Figure 1) predicted as responsible for binding were selected to study their relevance to binding and toxicity. The effects of the mutation of the selected Cry4Ba residues on the binding affinity to the Aa-mALP receptor were preliminarily determined using in silico mutation analysis using the BeAtMuSiC approach [24]. The BeAtMuSiC results revealed that all the alanine substitutions of the selected Cry4Ba residues decreased in stability of the Cry4Ba−Aa-mALP complex as ΔΔG_bind_ > 0 values were obtained (Figure 2).

### 2.2. Expression and Purification of Cry4Ba and Its Mutants

**Figure 3:**
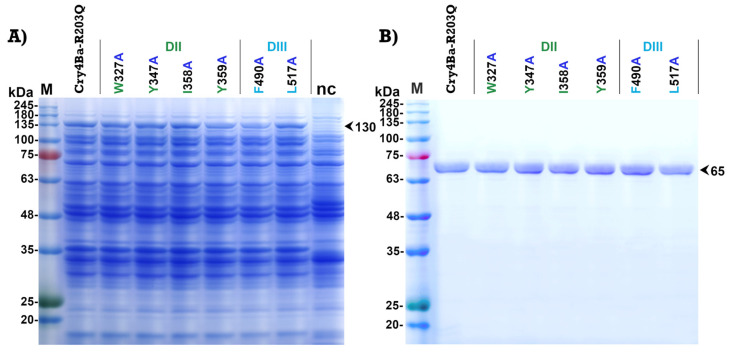
SDS-PAGE analysis (Coomassie brilliant blue-stained 10% gel) of (**A**) lysates extracted from *E. coli* (~10^7^ cells) expressing 130 kDa protoxins of Cry4Ba-R203Q or its domain II mutants (W327A, Y347A, I358A, and Y359A) and domain III mutants (F490A and L517A), or harboring the pUC12 vector (nc, a negative control) and (**B**) FPLC-purified 65 kDa trypsin-treated products of solubilized protoxins from (**A**). M represents the molecular mass standards.

The selected aromatic and hydrophobic residues (Trp_327_, Tyr_347_, Ile_358_, and Tyr_359_ on domain II and Phe_490_ and Leu_517_ on domain III) of Cry4Ba which were predicted as binding residues were individually mutated to alanine using PCR-based site-directed mutagenesis. All mutants were successfully constructed. After 4 h induction with isopropyl-β-D-thiogalactopyranoside (IPTG), all mutant Cry4Ba toxins were expressed as inclusion proteins in *E. coli* JM109 for which the sizes on polyacrylamide gel were 130 kDa (Figure 3A). The inclusion proteins of each mutant were purified from *E. coli* and solubilized in carbonate buffer, pH 9.2. Proteolytic activation of the solubilized protoxins by digestion with trypsin produced 65 kDa active fragments which were comparable to that of the Cry4Ba-R203Q toxin. The 65 kDa active toxins purified using a size-exclusion fast protein liquid chromatography (FPLC) system were shown in Figure 3B.

### 2.3. Larvicidal Activity of Cry4Ba and Its Mutants

**Figure 4:**
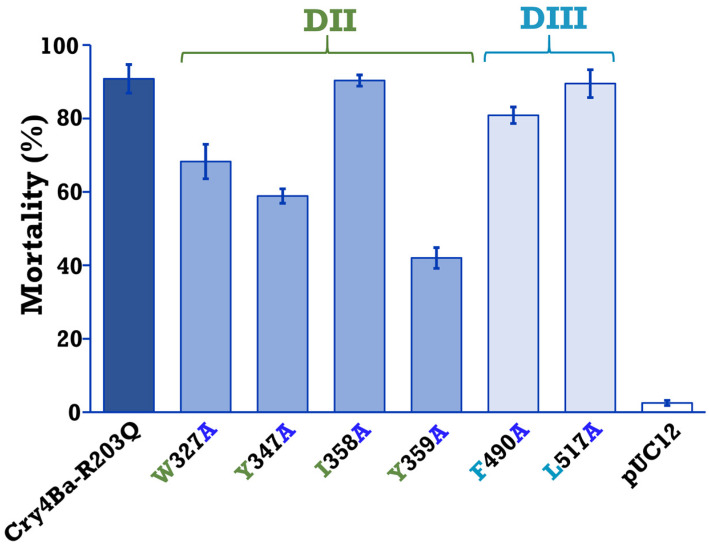
Mosquitocidal activity of *E. coli* (~10^8^ cells/mL) expressing 130 kDa protoxins of Cry4Ba-R203Q or its domain II (DII) mutants (W327A, Y347A, I358A, and Y359A) and domain III (DIII) mutants (F490A and L517A) against *A. aegypti* larvae. Cells harboring the pUC12 vector were used as a negative control. Error bars indicate standard error of the mean from three independent experiments.

The larvicidal activity of Cry4Ba and its mutants were assayed against 2-day-old *A. aegypti* larvae. Larval mortality was recorded after 24 h feeding *A. aegypti* larvae with *E. coli* cells expressing Cry4Ba-R203Q or its mutants. Bioassays revealed that the mutants W327A, Y347A, and Y359A of domain II exhibited significant decreases in toxicity (to 40–70%) (*p* < 0.003, Student’s *t*-test), with Y359A showing the highest toxicity reduction to approximately 40%, while the I358A, F490A, and L517A mutants retained their larvicidal activities (>80%) which were comparable to that of the Cry4Ba-R203Q toxin (Figure 4).

### 2.4. Expression and Purification of Aa-mALP and Its Mutants

**Figure 5:**
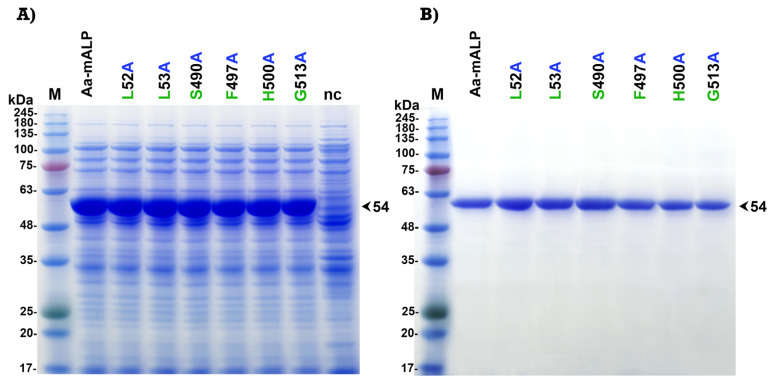
SDS-PAGE analysis (Coomassie brilliant blue-stained 10% gel) of (**A**) lysates extracted from *E. coli* (~10^7^ cells) expressing 54 kDa Aa-mALP or its mutants (L52A, L53A, S490A, F497A, H500A, and G513A) or harboring the pET-17b vector (nc, negative control) and (**B**) His-tag-fused Aa-mALP proteins from (**A**) purified using a Ni-NTA affinity column. M represents molecular mass standards.

The amino acid residues Leu_52_, Leu_53_, Ser_490_, Phe_497_, His_500_, and Gly_513_ of Aa-mALP with predicted binding with Leu_517_, Phe_490_, Tyr_347_, Tyr_359_, Ile_358_, and Trp_327_ of the Cry4Ba toxin, respectively, were individually mutated to alanine. All of the mutants were successfully generated and expressed as inclusion proteins in *E. coli* BL21 for which the sizes on polyacrylamide gel were 54 kDa (Figure 5A). The purified inclusion proteins were solubilized in PBS buffer containing 8 M urea, then purified and refolded in a nickel–nitrilotriacetic acid (Ni-NTA) affinity column. After desalting and buffer exchange, the Aa-mALP proteins were obtained in carbonate buffer (pH 9.2) with a size of 54 kDa on the gel (Figure 5B). To confirm that the purified Aa-mALP and its mutants were refolded as the native form, the alkaline phosphatase activity was analyzed based on dot blotting and detection with 5-bromo-4-chloro-3-indolyl phosphate/nitro blue tetrazolium (BCIP/NBT) chromogenic substrate (Supplementary Figure S1).

### 2.5. Quantitative Analysis of Cry4Ba Toxin—Aa-mALP Receptor Interactions

**Figure 6:**
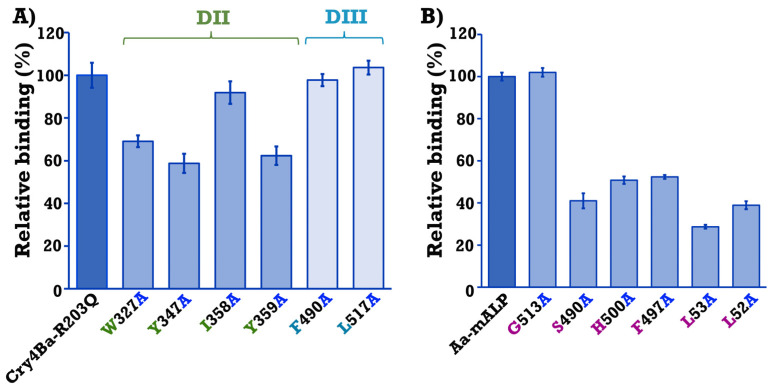
Relative binding activity of (**A**) Cry4Ba-R203Q and its mutants W327A, Y347A, I358A, and Y359A on domain II (DII) and F490A and L517A on domain III (DIII) against the Aa-mALP receptor. Binding of Cry4Ba-R203Q to the Aa-mALP receptor was taken as 100% and (**B**) Aa-mALP wild-type and its mutants G513A, S490A, H500A, F497A, L53A, and L52A against the Cry4Ba-R203Q toxin. Binding of wild-type Aa-mALP to the Cry4Ba-R203Q toxin was taken as 100%. Error bars indicate the standard error of the mean from three independent experiments.

The relevance on receptor binding of Trp_327_, Tyr_347_, Ile_358_, and Tyr_359_ on domain II and Phe_490_ and Leu_517_ on domain III of Cry4Ba was analyzed using ELISA. The quantitative binding studies revealed that mutation of aromatic residues on Cry4Ba domain II (W327A, Y347A, and Y359A) affected binding to the immobilized Aa-mALP receptor. The binding activities of these aromatic residues were significantly lower than for the Cry4Ba-R203Q toxin (*p* < 0.02, Student’s *t*-test) (Figure 6A). In contrast, the mutants I358A, F490A, and L517A did not affect binding to the Aa-mALP receptor, as shown in (Figure 6A).

Further characterization was performed by mutation of the Aa-mALP residues predicted to interact with the selected Cry4Ba residues and the effects of mutation on toxin–receptor interactions were studied. The residues Gly_513_, Ser_490_, His_500_, Phe_497_, Leu_53_, and Lue_52_ of Aa-mALP which interacted with Trp_327_, Tyr_347_, Ile_358_, Tyr_359_, Phe_490_, and Leu_517_ of the Cry4Ba toxin, respectively, were individually substituted with alanine and determined for interaction with the Cry4Ba-R203Q toxin using ELISA. The results revealed that the mutants S490A, H500A, F497A, L53A, and L52A affected binding with the toxin as significant decreases in their binding values were observed in the range 30–50% (*p* < 0.006, Student’s *t*-test), with L53A having the highest decrease of approximately 30%, while the G513A mutant retained its binding to the toxin that was comparable to that of the wild-type Aa-mALP receptor (Figure 6B).

## 3. Discussion

Receptor binding is a prerequisite step to exert the larvicidal activity of Cry toxins [25]. Domains II and III of Cry toxins have been reported to function in binding to receptors on epithelial cells of the larval midgut (for review, see [26]). For the Cry4Ba toxin, several amino acid residues on β-hairpin loops, especially those located on the lower part of domain II such as Thr_386_, Ser_387_, Ser_388_, Pro_389_, Ser_390_, and Asn_391_ on the β6–β7 loop, Glu_417_ on the β8–β9 loop, and Tyr_455_ and Asn_456_ on the β10–β11 loop, have been reported to be important for binding and toxicity [16,22]. Our previous study [21], using molecular docking, revealed a possible Cry4Ba toxin—Aa-mALP receptor interaction in which surface residues on the side of the domain II, such as residues on β2 and β4 and residues on the β-hairpin loops of domains II and III rather than only the residues on the β-hairpin loops at the bottom of domain II, are responsible for binding to the Aa-mALP receptor (Figure 1). According to a previous study, three fundamental hotspot residues (tryptophane, tyrosine, and arginine) were reported to contribute more significantly to binding affinity in protein–protein interfaces compared with others (for review, see [27]). In addition, hydrophobic residues were reported to be important for protein–protein interactions [21,28]. In the present work, some aromatic and hydrophobic residues on the surface of Cry4Ba domain II (Trp_327_, Tyr_347_, Ile_358_, and Tyr_359_) and domain III (Phe_490_ and Leu_517_) that were predicted as binding residues were therefore selected to study their relevance to the binding and toxicity of the Cry4Ba toxin. In silico analysis of the change in binding free energy (ΔΔG_bind_) of a protein–protein complex upon mutation of the amino acid residue to alanine using the BeAtMuSiC approach revealed moderate destabilization change (ΔΔG_bind_~1.5–2.0 kcal/mol), suggesting that these residues may be essential for binding to the Aa-mALP receptor. The ΔΔG_bind_ values analyzed using BeAtMuSiC have been reported to be more than 0.5 kcal/mol of alanine substitution of residues crucial for bioactivity [21,29].

PCR-based site-specific mutagenesis was performed to substitute Trp_327_, Tyr_347_, Ile_358_, Tyr_359_, Phe_490_, and Leu_517_ of Cry4Ba residues to alanine. All of the Cry4Ba mutant proteins were heterologously expressed in *E. coli* at similar levels compared to the Cry4Ba-R203Q protein and their tryptic digestion patterns were comparable with that of the Cry4Ba-R203Q toxin, implying that the mutations did not affect folding of the mutant proteins. Protease sensitivity change was reported to be caused by structural alteration by mutation of Cry toxins [30]. Larvicidal activity assays against *A. aegypti* showed a significant decrease in toxicity of aromatic residue mutations on Cry4Ba domain II (W327A, Y347A, Y359A), suggesting that these aromatic residues, especially Tyr_359_ which had a more adverse effect on toxicity, may be related to receptor binding. The involvement in receptor binding of the aromatic residues Trp_327_, Tyr_347_, and Tyr_359_ of Cry4Ba domain II was supported by Cry4Ba-Aa-mALP binding assays using ELISA that showed a decrease in binding of W327A, Y347A, and Y359A to the Aa-mALP receptor which correlated with their larvicidal activities. Notably, not all of the predicted binding residues were involved in the binding and toxicity of the toxin. However, the decreases in both the receptor binding and toxicity of the Cry4Ba mutants W327A, Y347A, and Y359A implied an important role of these aromatic residues.

Even the Cry4Ba mutants I358A, F490A, and L517A retained their toxicity and binding to the Aa-mALP receptor, while their counterpart Aa-mALP mutant residues H500A, L53A, and L52A showed significant decreases in binding to the toxin. This may have been due to the binding of His_500_, Leu_53_, and Lue_52_ of Aa-mALP residues to more than one binding residue of the Cry4Ba toxin; therefore, a single mutation of these residues provided more effect on binding activities. For example, the Cry4Ba-F490A mutant retained binding to the wild-type Aa-mALP receptor, while its counterpart Aa-mALP-L53A mutant showed a large decrease in binding to the toxin that may have been due to Lue_53_ bonding not only to Phe_490_ but also to Arg_52_ and Glu_522_ of the toxin.

**Figure 7:**
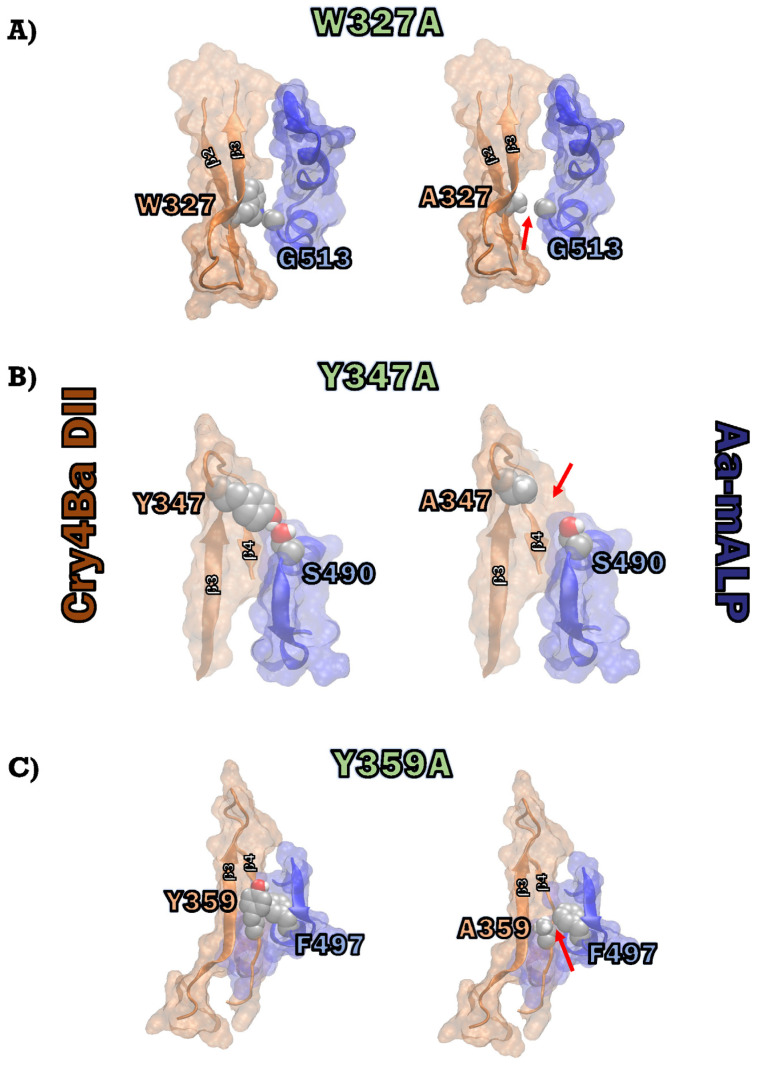
Surface-ribbon representation of part of docking conformations of Cry4Ba—Aa-mALP interaction prepared using VMD software [23]: (**A**) hydrophobic interaction between Cry4Ba DII-Trp_327_ and the backbone carbon of Aa-mALP-G_513_ (**left panel**) along with decreased contact (denoted by red arrow) resulting from W327A mutation (**right panel**); (**B**) hydrogen-bonding interaction between Cry4Ba DII-Tyr_347_ and Aa-mALP-Ser_490_ (**left panel**) along with diminished contact (denoted by red arrow) in the Y347A mutation (**right panel**); (**C**) hydrophobic interaction between Cry4Ba DII-Tyr_359_ and Aa-mALP-Phe_497_ (**left panel**) along with decreased contact (denoted by red arrow) resulting from Y359A mutation (**right panel**).

Tryptophane is one of three top-range hot-spot residues in protein–protein interfaces; the Trp/Ala mutation was reported to create a large cavity due to its large size and aromatic nature [31]. The presence of conservative Trp on the protein surface was reported as indicating a highly possible binding site [31]. Mutation W327A may affect Cry4Ba toxin—Aa-mALP receptor interaction and hence toxicity by disruption of the hydrophobic interaction of the aromatic side chain of Trp_327_ on the β2 strand of the Cry4Ba toxin and the backbone of Gly_513_ of the Aa-ALP receptor (Figure 7A). Mutation by Gly513Ala of the Aa-ALP receptor that did not affect the binding of the toxin may support the binding of Trp_327_ to the backbone of Gly_513_. Tyrosine was one of the enriched amino acids acting as a hot-spot residue in protein–protein interactions that primarily contributed hydrogen-bonding with polar and charge residues or interaction with backbone atoms and sidechain carbon [32,33]. Mutation Y347A eliminated the hydrogen-bonding between Tyr_347_ on the β3–β4 loop of the Cry4Ba toxin and Ser_490_ of the Aa-mALP receptor (Figure 7B), which may have been a cause of decreased receptor binding and hence toxicity of the toxin. The binding interaction Tyr_347_-Ser_490_ was also supported by the decreased binding of the Aa-mALP-S490A mutant to the Cry4Ba toxin. Mutation Y359A may affect the binding and toxicity of the Cry4Ba toxin by interrupting the hydrophobic interaction between Tyr_359_ on the β4 strand of the Cry4Ba toxin and Phe_497_ of the Aa-mALP receptor (Figure 7C). The binding interaction of Tyr_359_-Phe_497_ was also supported by the decreased binding of the Aa-mALP-F497A mutant to the Cry4Ba toxin.

Aromatic amino acids, especially Tyr, have been reported to play an important role in receptor binding which is a prerequisite for the toxicity of many toxins, such as the cytolethal distending toxin (Cdt) of *Aggregatibacter actinomycetemcomitans* [34], the alpha toxin (AT) of *Clostridium septicum* [35], and the binary toxin (Bin) of *Bacillus sphaericus* [36]. It is possible that the aromatic residues Trp_327_, Tyr_347_, and Tyr_359_ on the side surface of Cry4Ba-domain II are required for binding with the Aa-mALP receptor to exhibit larvicidal activity.

Previous reports mainly focused on the exploration of amino acid residues on β-hairpin loops located on the lower part of the Cry4Ba domain II function in receptor-binding [16,22], whereas the present study investigated the binding function of residues on β strands and β-hairpin loops on the side of the Cry4Ba toxin. We clearly showed that the aromatic residues Trp_327_ on the β2 strand, Tyr_347_ on the β3–β4 loop, and Tyr_359_ on the β4 strand of the Cry4Ba-domain II were involved in binding to the Aa-mALP receptor and hence, toxicity. The finding in this work would be helpful to understand the receptor-binding mechanism of the Cry4Ba toxin.

## 4. Conclusions

In conclusion, we identified that the aromatic residues Trp_327_ on the β2 strand, Tyr_347_ on the β3–β4 loop, and Tyr_359_ on the β4 strand of the Cry4Ba toxin played essential roles in binding to the Aa-mALP receptor. Our work highlighted the importance of the side surface residues of the Cry4Ba molecule on receptor binding which provides greater insight into the possible mechanism of the Cry4Ba toxin. Further protein engineering could be applied to achieve a more potent toxin to control the mosquito vectors of dengue viruses.

## 5. Materials and Methods

### 5.1. Materials

Mutagenic primers designed to a specific mutation at selected residues of the Cry4Ba toxin and the Aa-mALP receptor were purchased from Macrogen (Seoul, Republic of Korea). *pfu* DNA polymerase, restriction enzymes, and isopropyl-β-D-thiogalactopyranoside (IPTG) were purchased from Vivantis Technologies (Selangor, Malaysia). Nickel–nitrilotriacetic acid (Ni-NTA) affinity columns and tolylsulfonyl phenylalanyl chloromethyl ketone (TCPK)-treated trypsin were purchased from Thermo Fisher Scientific (Rockford, IL, USA). The rabbit anti Cry4Ba antibody was kindly provided by the Bacterial Toxin Research Innovation Cluster (BRIC), Institute of Molecular Biosciences, Mahidol University, Thailand. The 96-well maxi-binding immunoplates were purchased from SPL Life Science (Gyeonggi, Republic of Korea). Horse radish peroxidase (HRP)-conjugated goat anti-rabbit IgG and the 3,3,5,5-tetramethylbenzidine (TMB) substrate were purchased from Cell Signaling Technology (Beverly, MA, USA). Alkaline phosphatase chromogenic substrate 5-bromo-4-chloro-3-indolyl phosphate/nitro blue tetrazolium (BCIP/NBT) was purchased from Sigma-Aldrich (St Louis, MO, USA). PD-10 desalting and 10 kDa Vivaspin concentrator columns were purchased from Cytiva (Uppsala, Sweden). Bradford’s reagent was purchased from HiMedia Laboratories (Maharashtra, India). *Aedes aegypti* eggs were purchased from the Department of Medical Sciences, Ministry of Public Health of Thailand (Bangkok, Thailand). Unless otherwise indicated, all other reagents were of analytical grade.

### 5.2. In Silico Binding Analysis of Cry4Ba Toxin—Aa-mALP Receptor Interaction

The in silico binding interaction between the active form of the Cry4Ba toxin (PDB:1W99) and the homology modeled Aa-mALP using ClusPro 2.0 molecular docking software was obtained from our previous work [21]. Interacting residues were analyzed using LIGPLOT 1.4.4 software [37]; then, aromatic and hydrophobic residues on the surface of Cry4Ba domains II and III predicted as being responsible for binding were selected for further site-specific mutagenesis. A possible effect of alanine substitution of possible binding residues of Cry4Ba on toxin–receptor interactions was preliminarily analyzed by computing the binding affinity changes (ΔΔG_bind_) using the in silico mutation analysis BeAtMuSiC approach [24]. The BeAtMuSiC software calculated the binding free energy based on statistical potentials, where the mutation is destabilizing if ΔΔG_bind_ > 0, while when ΔΔG_bind_ < 0, the respective mutation is stabilizing [38].

### 5.3. Construction of Cry4Ba Mutant Plasmids

Six aromatic and hydrophobic residues (Trp_327_, Tyr_347_, Ile_358_, and Tyr_359_ on domain II, and Phe_490_ and Leu_517_ on domain III of the Cry4Ba toxin) predicted as potential binding residues were selected and individually substituted with alanine (alanine scanning) using the Quick Change site-directed mutagenesis procedure developed by Stratagene (La Jolla, USA). Mutagenic primers were designed based on the nucleotide sequence of the *cry4Ba* gene (NCBI accession number X07423) to substitute each selected residue with alanine (Supplementary Table S1). A p4Ba-R203Q plasmid, encoding 130 kDa Cry4Ba-R203Q (in which one trypsin-sensitive residue (Arg_203_) was mutated to Gln to produce a 65 kDa activated toxin for easy purification as described elsewhere [39]), was used as a template for polymerase chain reaction (PCR)-based site-directed mutagenesis by the activity of *pfu* DNA polymerase. Mutagenized plasmids were transformed into *E. coli* strain JM109. Mutant clones were primarily identified based on digestion with restriction endonuclease and subsequently confirmed using DNA sequencing from the commercial services of First BASE Laboratories (Selangor, Malaysia).

### 5.4. Construction of Aa-mALP Mutant Plasmids

Six potential binding residues of the Aa-mALP receptor (Leu_52_, Leu_53_, Ser_490_, Phe_497_, His_500_, and Gly_513_) with predicted binding with Leu_517_, Phe_490_, Tyr_347_, Tyr_359_, Ile_358_, and Trp_327_ of the Cry4Ba toxin, respectively, were individually substituted with alanine using the method described above. Mutagenized primers (Supplementary Table S1) were designed according to the nucleotide sequence of the alkaline phosphatase gene from the *A. aegypti* midgut (NCBI accession number GQ395622). A pET-Aa-mALP plasmid encoding C-terminal His-tagged Aa-mALP under the control of the T7 promoter [20] was used as a template for site-directed mutagenesis. Mutagenized plasmids were transformed into *E. coli* strain BL21(DE3). Mutant clones were identified as described above.

### 5.5. Expression and Purification of Cry4Ba and Its Mutants

The 65 kDa active Cry4Ba toxins were prepared as described previously [40]. In brief, 130 kDa Cry4Ba-R203Q and its mutants protoxins were overexpressed as cytoplasmic inclusions in *E. coli* JM109 upon induction with 0.1 mM IPTG for 4 h. After solubilization in carbonate buffer (50 mM Na_2_CO_3_/NaHCO_3_ (pH 9.2)) for 1 h, the protoxins were activated by digestion with TCPK-treated trypsin (1:20, *w*/*w*) for 16 h into 65 kDa active toxins. The 65 kDa trypsin-activated toxins were purified using a size exclusion fast protein liquid chromatography system on a Superdex-200 HR column from Amersham-Pharmacia Biotech (Piscataway, NJ, USA) as described previously [22] and then concentrated using a Vivaspin concentrator column (10 kDa MWCO). The purified proteins were determined for their concentrations based on Bradford assay and analyzed using sodium dodecyl sulfate polyacrylamide gel electrophoresis (SDS-PAGE).

### 5.6. Expression and Purification of Aa-mALP and Its Mutants

The 54 kDa Aa-mALP and its mutants were prepared as described previously [20]. In brief, upon induction with 0.1 mM IPTG for 4 h, the His-tag-fused Aa-ALP proteins were overexpressed as inclusion in *E. coli* BL21. Inclusions were solubilized for 1 h at 25 °C in phosphate-buffered saline (PBS) containing 8 M urea, pH 7.5. Purification and refolding were performed in a Ni-NTA affinity column using gradients of decreasing urea concentrations and elution with PBS containing 500 mM imidazole, pH 7.5. Buffer exchange into a carbonate buffer (pH 9.2) was performed on a PD-10 desalting column according to the manufacturer’s protocol. The concentrations of purified Aa-mALP and its mutant proteins were determined using Bradford assay and then analyzed by SDS-PAGE.

### 5.7. Mosquito Larvicidal Activity Assays

The mosquitocidal activity of the Cry4Ba-R203Q and its mutants was investigated against two-day-old *A. aegypti* larvae. The assays were carried out in 48-well cell culture plates at 30 °C. Each well contained 10 larvae in 1 mL of cell suspension (10^8^ cells of *E. coli* expressing Cry4Ba-R203Q toxin or its mutants in ddH_2_O) and a total of 100 larvae were used to assay for each toxin. *E. coli* containing a pUC19 plasmid without the insecticidal toxin gene was used as a negative control. Three independent replications were performed for each treatment.

### 5.8. Cry4Ba Toxin-Aa-mALP Binding Assays Using ELISA

Binding interactions between the Cry4Ba toxin and the Aa-mALP receptor were analyzed using ELISA, as described previously with some modifications [21]. The purified Aa-ALP receptor or its mutants were coated (2.5 μg in 200 μL of 50 mM carbonate buffer, pH 9.2) to 96-well maxi-binding immunoplates at 4 °C for 4 h. Then, after washing five times with PBS containing 0.02% Tween 20 (PBS-T), pH 7.4, and blocking with 5% (*w*/*v*) skimmed milk in PBS-T, pH 7.4 for 1 h, the coated wells were incubated with 100 nM of each purified Cry4Ba-R203Q, its mutants, or a negative control ligand–bovine serum albumin at 37 °C for 2 h. The Cry4Ba toxin proteins bound to the immobilized Aa-mALP receptor were detected based on incubation with rabbit anti-Cry4Ba antibodies (1:10,000 dilution) for 1 h, followed with HRP-conjugated goat anti-rabbit IgG (1:5000) for 1 h. The TMB substrate was added to the wells and incubated for 10 min. The reaction was stopped by the addition of 1 N HCl and absorbance at 450 nm was measured using a Multiskan ELISA plate reader (Thermo Fisher Scientific, Rockford, IL, USA).

### 5.9. Statistical Analysis

Each experiment was performed at least three independent times. Statistics were calculated using the Student’s *t*-test to analyze significant differences between the wild-type and each mutant. A *p* value < 0.05 was considered statistically significant.

## Data Availability

Not applicable.

## Supplementary Materials

The following supporting information can be downloaded at: https://www.mdpi.com/article/10.3390/toxins15020114/s1, Figure S1: Alkaline phosphatase activities of purified Aa-mALP wild-type and its mutants. Table S1: Mutagenized primers for alanine substitution of selected amino acid residues on domains II and III of the Cry4Ba toxin and the Aa-mALP receptor.
