# Supplementary material for: Aromatic Residues on the Side Surface of Cry4Ba-Domain II of Bacillus thuringiensis subsp. israelensis Function in Binding to Their Counterpart Residues on the Aedes aegypti Alkaline Phosphatase Receptor
Source: Toxins (Basel). 2023 Jan 29;15(2):114. doi: 10.3390/toxins15020114 (PMC9960242; doi:10.3390/toxins15020114)
Supplement: Supplementary file 1 [file toxins-15-00114-s001.zip › Supplementary Figure S1.pdf]

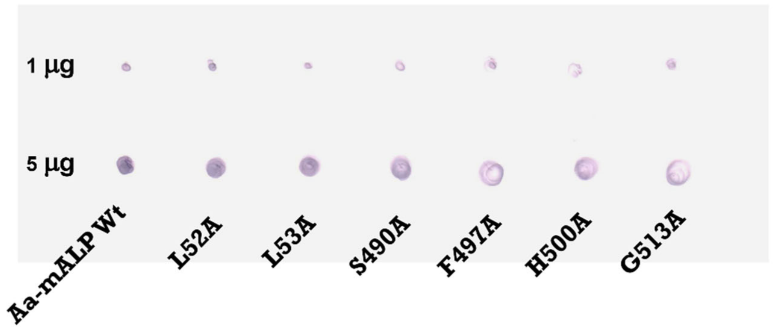


**Supplementary Figure S1** Alkaline phosphatase activities of purified Aa-mALP wild-type and its mutants.
