# Supplementary material for: Aromatic Residues on the Side Surface of Cry4Ba-Domain II of Bacillus thuringiensis subsp. israelensis Function in Binding to Their Counterpart Residues on the Aedes aegypti Alkaline Phosphatase Receptor
Source: Toxins (Basel). 2023 Jan 29;15(2):114. doi: 10.3390/toxins15020114 (PMC9960242; doi:10.3390/toxins15020114)
Supplement: Supplementary file 1 [file toxins-15-00114-s001.zip › Supplementary Table S1.pdf]

Supplementary Table S1. Mutagenized primers for alanine substitution of selected amino acid residues on domains II and III of Cry4Ba toxin and Aa-mALP receptor.

Protein	Primer	Sequence (5′ ==> 3′)	Restriction site
Cry4Ba domain II	W327A-f	GAGTAGA**C**TTC**GC**GACCAATACTATATA	*Acc*I
	W327A-r	ATTGGTC**GC**GAA**G**TCTACTCTCTTTAG	
	Y347A-f	TGGGTT**C**TCA**GC**TACAAATTCTTCTGCA	*Dde*I
	Y347A-r	AATTTGTA**GC**TGA**G**AACCCAATTTTATTGG	
	I358A-f	AAGTGGA**GCA**TATGGAAGTTCTGG	*Nde*I
	I358A-r	TTCCATA**TGC**TCCACTTTCTTGCAT	
	Y359A-f	TGGAATT**GC**TGGA**TCC**TCTGGTTTTGGTTC	*Bam*HI
	Y359A-r	ACCAGA**GGA**TCCA**GC**AATTCCACTTTCTTG	
Cry4Ba domain III	F490A-f	AATCTAAC**GCT**TT**A**AATGCAACAGCTAAA	*Dra*I
	F490A-r	TTGCATT**T**AA**AGC**GTTAGATTTTACGGCC	
	L517A-f	TGGTAC**CGC**ATCAGGCAGAATGG	*Kpn*I
	L517A-r	CCTGAT**GCG**GTACCATTGCTTGTA	
Aa-mALP	L52A-f	GCACGAT**GC**CTTATT**C**GA**A**AAAAAGGATTATACGA	*Bst*BI
	L52A-r	TCCTTTTT**T**T**C**GAATAAG**GC**ATCGTGCGCCTG	
	L53A-f	GATCTC**GC**ATT**C**GA**A**AAAAAGGATTATACGA	*Bst*BI
	L53A-r	CCTTTTT**T**TC**G**AAT**GC**GAGATCGTGCGC	
	S490A-f	TCATGTTT**G**CCGGTGG**C**TACGAGCAAAACTTC	*Bci*VI
	S490A-r	CTCGTA**G**CCACCGG**C**AAACATGAACGCCC	
	F497A-f	GCAAAAC**GCG**AT**C**GCCCATGCCATC	*Bsp*143I
	F497A-r	ATGGGC**G**AT**CGC**GTTTTGCTCGTATC	
	H500A-f	TCATTGC**TGCA**GCCATCTCGTTTG	*Pst*I
	H500A-r	GATGGC**TGCA**GCAATGAAGTTTTGC	
	G513A-f	TGTGTG**C**AGG**A**GC**T**CACCACCACCAC	*Sac*I
	G513A-r	GTGGTG**A**GC**T**CCT**G**CACACACGTCCG	

Bold letters indicate mutated nucleotide residues; f and r represent forward and reverse primers, respectively. Underlined and double underlined bases represent the recognition sites introduced and eliminated for restriction analysis, respectively.
